# The T6SSs of *Pseudomonas aeruginosa* Strain PAO1 and Their Effectors: Beyond Bacterial-Cell Targeting

**DOI:** 10.3389/fcimb.2016.00061

**Published:** 2016-06-09

**Authors:** Thibault G. Sana, Benjamin Berni, Sophie Bleves

**Affiliations:** ^1^Laboratoire d'Ingénierie des Systèmes Macromoléculaires (UMR7255), IMM, Centre National de la Recherche Scientifique and Aix-Marseille UniversityMarseille, France; ^2^Department of Microbiology and Immunology, Stanford School of Medicine, Stanford UniversityStanford, CA, USA

**Keywords:** *Pseudomonas aeruginosa*, Type Six Secretion System, invasion mechanism, epithelial cells, gamma-tubulin complex, microtubules, PI3K Akt pathway, antibacterial activity

## Abstract

*Pseudomonas aeruginosa* is an opportunistic pathogen responsible for many diseases such as chronic lung colonization in cystic fibrosis patients and acute infections in hospitals. The capacity of *P. aeruginosa* to be pathogenic toward several hosts is notably due to different secretion systems. Amongst them, *P. aeruginosa* encodes three Type Six Secretion Systems (T6SS), named H1- to H3-T6SS, that act against either prokaryotes and/or eukaryotic cells. They are independent from each other and inject diverse toxins that interact with different components in the host cell. Here we summarize the roles of these T6SSs in the PAO1 strain, as well as the toxins injected and their targets. While H1-T6SS is only involved in antiprokaryotic activity through at least seven different toxins, H2-T6SS and H3-T6SS are also able to target prokaryotic as well as eukaryotic cells. Moreover, recent studies proposed that H2- and H3-T6SS have a role in epithelial cells invasion by injecting at least three different toxins. The diversity of T6SS effectors is astounding and other effectors still remain to be discovered. In this review, we present a table with other putative *P. aeruginosa* strain PAO1 T6SS-dependent effectors. Altogether, the T6SSs of *P. aeruginosa* are important systems that help fight other bacteria for their ecological niche, and are important in the pathogenicity process.

The Type Six Secretion system (T6SS) was discovered ten years ago in the laboratory of Pr. J. Mekalanos (Mougous et al., 2006; Pukatzki et al., 2006). It functions as a contractile molecular syringe consisting of a sheath and a puncturing device made of an Hcp tube terminated by a spike of VgrG and PAAR proteins. Contraction of the TssB/C sheath propels the puncturing device out of the cell into a target cell and leads to the injection of effector proteins (Alcoforado Diniz et al., 2015). The first studies focused on the phenotypes associated with T6SS mutations in different pathogens in the context of eukaryotic host infection. However in 2010, Dr. J. Mougous laboratory showed an unsuspected antibacterial activity mediated by the H1-T6SS from *Pseudomonas aeruginosa*, making T6SSs transkingdom machineries (Hood et al., 2010). Since this discovery, T6SSs have mainly been studied for their capacity to target prokaryotes and this would seem to be their primary function. But interestingly, several T6SSs are also known to target both prokaryotes and eukaryotes such as the T6SS of *Vibrio cholera* (Pukatzki et al., 2006; MacIntyre et al., 2010).

*P. aeruginosa* is one of the most virulent opportunistic human pathogens and is responsible for many diseases such as broncho-alveolar colonization in cystic fibrosis patients or acute infections of lungs and burned skin that can lead to septicemia. Its genome encodes many virulence factors including several secretion systems that help *P. aeruginosa* control its environment and the activity of host cells (Bleves et al., 2010). Among theses, *P. aeruginosa* harbors three independent Type Six Secretion Systems (T6SS). This review will aim to describe the roles, effectors, and targets of these three T6SSs.

## *P. aeruginosa* uses T6SSs as antiprokaryotic weapons

T6SSs are present in more than 200 Gram-negative bacteria including *P. aeruginosa*, whose genome encodes three different T6SS loci named H1-, H2-, and H3-T6SS (Table 1). Historically, H1-T6SS is the first T6SS machinery that was shown to display an antibacterial activity (Hood et al., 2010). H1-T6SS serves as a counter-attack weapon to outcompete other T6SS^+^ bacteria that coexist in a same ecological niche, and confers a growth advantage upon *P. aeruginosa* (Basler et al., 2013). More specifically, *P. aeruginosa* targets other bacteria through H1-T6SS dependent injection of effector Tse2 and also produces an anti-toxin Tsi2, protecting itself against the intrinsic effect of the toxin and from attack by sister-cells (Hood et al., 2010). It was recently shown that Tse2 induces quiescence in bacterial target cells, and that Tsi2 directly interacts with Tse2 in the cytoplasm to inactivate its lethal activity (Li et al., 2012). Recently, Tse2 toxicity was shown to be NAD-dependent and may involve an ADP-ribosyltransferase activity (Robb et al., 2016). Besides Tse2, which acts in the cytoplasm of prey cells, Tse1 and Tse3 are injected into the periplasm of target bacterial cells through H1-T6SS (Russell et al., 2011). Tse1 and Tse3 hydrolyse peptidoglycan, providing a fitness advantage for *P. aeruginosa* in competition with other bacteria. To protect from killing by sister-cells, *P. aeruginosa* uses the periplasmic immunity proteins Tsi1 and Tsi3 which counteract Tse1 and Tse3 toxicity (Russell et al., 2011). Later, X-ray studies revealed that Tse1 cleaves the γ-D-glutamyl-l-meso-diaminopimelic acid amide bond of crosslinked peptidoglycan (Benz et al., 2012; Chou et al., 2012). Moreover, the crystal structure of Tse1 in interaction with Tsi1 demonstrates that the immunity protein occludes the active site of Tse1 abolishing its enzyme activity (Benz et al., 2012). Tse3 functions as a muramidase, cleaving the β-1,4-linkage between N-acetylmuramic acid and N-acetylglucosamine in peptidoglycan (Lu et al., 2013). These three effectors were discovered in 2010 thanks to their coregulation with the H1-T6SS machinery (Table 1), and other H1-T6SS toxins were described later. Tse4 was identified as a H1-T6SS effector using quantitative cellular proteomics in interaction with Hcp (Whitney et al., 2014). Tse5, Tse6, and Tse7 were identified by their genetic association with VgrGs and the H1-T6SS (Hachani et al., 2014; Whitney et al., 2014). Those four effectors display antibacterial activity and are associated with cognate immunities (Table 1). Recently Tse6 was shown to degrade the essential dinucleotides NAD(+) and NADP(+) leading to bacteriostasis in the target bacterium (Whitney et al., 2015). Intriguingly Tse6 delivery into the host cytoplasm requires translation elongation factor Tu (EF-Tu). The interaction of a toxin with a house keeping protein may suggest that it can target phylogenetically diverse bacteria (Whitney et al., 2015). EF-Tu may facilitate Tse6 translocation into recipient cells or by driving the H1-T6SS needle at the cell surface of the preys either by favoring the passage of the toxin once delivered into the periplasm to the cytoplasm. Indeed EF-Tu is known as a moonlighting protein or anchorless multifunctional protein that is capable, when localized to the cell surface, of interfering with bacterial adherence (for a review see Henderson and Martin, 2011). Importantly work done on H1-T6SS toxins has revealed different conserved mechanisms for targeting T6SS effectors to the T6SS machinery (Table 1): (i) Hcp-dependent recruitment in the case of Tse1-4, (ii) direct VgrG-targeting for Tse5, (iii) VgrG-targeting for Tse7 through a PAAR motif and for Tse6 through an adaptator/chaperonne protein called EagT6. Altogether, H1-T6SS is a formidable antibacterial weapon, injecting many different effectors to compete bacterial cells, and allowing *P. aeruginosa* to overwhelm them during competition for the same ecological niche.

**Table 1:** **Immunity proteins, enzymatic activities, targets, localizations, and recruitment of the T6SS effectors of the *P. aeruginosa* strain PAO1**.

**PAO1 Effector**	**PA number**	**Immunity**	**Target-cell**	**Host-cell localization**	**Regulation**	**Activity**	**Activity/Function**	**Recruitment to the T6SS machinery**	**References**
H1-T6SS					RetS repression QS repression				Mougous et al., 2006; Lesic et al., 2009
Tse1 (Type six exported)	PA1844	Tsi1(Type six immunity) (PA1845)	Bacteria	Periplasm	RetS repression	Amidase	Peptidoglycan degradation	Hcp1 (PA0085)- dependent	Hood et al., 2010; Russell et al., 2011; Benz et al., 2012; Chou et al., 2012; Silverman et al., 2013
Tse2	PA2702	Tsi2(PA2703)	Bacteria	Cytoplasm	RetS repression	NAD dependent toxicity	Bacteriostatic	Hcp1 (PA0085)- dependent	Hood et al., 2010; Li et al., 2012; Robb et al., 2016; Silverman et al., 2013
Tse3	PA3484	Tsi3(PA3485)	Bacteria	Periplasm	RetS repression	Muramidase	Peptidoglycan degradation	Hcp1 (PA0085)- dependent	Hood et al., 2010; Russell et al., 2011; Lu et al., 2013; Silverman et al., 2013
Tse4	PA2774	Tsi4 (PA2775)	Bacteria	Periplasm		?	4 transmembrane segments	Hcp1 (PA0085)- dependent	Whitney et al., 2015
Tse5 (RhsP1)	PA2684	Tsi5 (PA2684.1)	Bacteria	Periplasm/ membrane	RetS repression	?	RHS/YD repeat, toxicity	VgrG1c (PA2685)- dependent	Hachani et al., 2014; Whitney et al., 2014
Tse6	PA0093	Tsi6 (PA0092)	Bacteria	Cytoplasm		NAD(P)+ glycohydrolase	Bacteriostatic, NAD(P)+ depletion, PAAR motif	VgrG1a (PA0091)- dependent trhough a PAAR motif & the EagT6 (effector-associated gene with Tse)(PA0094) chaperone	Alcoforado Diniz et al., 2015; Whitney et al., 2015
Tse7	PA0099	?	Bacteria	Cytoplasm?		Endonuclease?	TOX-GHH2 signature	VgrG1b (PA0095)-dependent	Hachani et al., 2014
H2-T6SS					QS activation Fur repression PsrA repression Exponential phase RpoN repression				Kang et al., 2008; Siehnel et al., 2010; Sana et al., 2012, 2013
PldA (Tle5a) (Type six lipase effector)	PA3487	Tli5a (PA3488) (Type six lipase immunity)	Bacteria eukaryote	Periplasm cytosol		Phospholipase D	Cell wall integrity internalization through Akt binding	VgrG4b (PA3486)- dependent?	Wilderman et al., 2001; Russell et al., 2013; Jiang et al., 2014; Spencer and Brown, 2015
VgrG2b	PA0262		Eukaryotes	Cytosol	Exponential phase	Protease?	γ-TurC and microtubule-dependent internalization	Evolved VgrG	Sana et al., 2015
**H2-T6SS Putative**									
Tle1	PA3290	Tli1 (PA3291)	Bacteria	Periplasm		Phospholipase A2	Toxicity	VgrG4a (PA3294)- dependent trhough a chaperonne (PA3293) with a DUF4123?	Barret et al., 2011; Russell et al., 2013;Hu et al., 2014; Alcoforado Diniz et al., 2015
Tle3	PA0260	Tli3(PA0259)	Bacteria	Periplasm		Lipolytic	Toxicity	VgrG2b (PA0262)- dependent?	Barret et al., 2011; Russell et al., 2013; Sana et al., 2015
Tle4	PA1510	Tli4 (PA1509)	Bacteria	Periplasm		Lipolytic	Toxicity	VgrG2a (PA1511)- dependent?	Barret et al., 2011; Lu et al., 2013; Russell et al., 2013; Sana et al., 2015
PA1508	PA1508	?	?	?		?	PAAR motif	VgrG2a (PA1511)- dependent trhough a PAAR motif?	This review
H3-T6SS					QS activation RpoN Stationary phase				Lesic et al., 2009; Sana et al., 2013
PldB (Tle5b)	PA5089	Tli5b1(PA5086) Tli5b2(PA5087) Tli5b3(PA5088)	Bacteria eukaryote	Periplasm cytosol	Stationary phase	Phospholipase D	Cell wall integrity internalization through Akt binding	VgrG5 (PA5090)- dependent?	Russell et al., 2013; Jiang et al., 2014

*Putative effectors are highlighted in light blue. Green is H1-T6SS, dark blue is H2-T6SS, and orange is H3-T6SS. Related effectors are listed below until new color and new secretion is described. Light blue are the putative effectors of H2-T6SS*.

H2-T6SS and H3-T6SS also display antibacterial activity by injecting the phospholipase D enzymes PldA and PldB that belong to the Tle5 (type VI lipase effector) family into other bacterial cells (Russell et al., 2013; Jiang et al., 2014). The PldA toxin functions by degrading the major constituent of bacterial membranes, phosphatidylethanolamine (Russell et al., 2013). However, despite strong evidence that *P. aeruginosa* T6SSs participate widely in bacterial competition, several recent reports have focused on the ability of H2 and H3-T6SS to target epithelial cells.

## *P. aeruginosa* T6SSs also target eukaryotic cells

So far, H1-T6SS has never been shown to be directly involved in anti-eukaryotic activity. However, Hcp1 has been found in pulmonary secretions of cystic fibrosis patients as well as Hcp1-specific antibodies in their sera (Mougous et al., 2006), suggesting that the antiprokaryotic activity of H1-T6SS could be necessary for host colonization in a complex microbial community. Interestingly, several members of the gut microbiota actually encode T6SSs that could lead to the contact-dependent killing of other bacteria, including *Bacteroidetes fragilis* (Russell et al., 2014). This opens a new field of research at the interface between the pathogen, host and microbiota, giving a protective role for the commensal microbiota through T6SS-dependent killing of the pathogen. In support of this hypothesis, *V. cholerae* needs some of its antitoxins to establish in the host gut, strongly suggesting that it is subject to T6SS attacks from the microbiota (Fu et al., 2013). Moreover, it was shown that about half of the *Bacteroidales* genomes, the most prevalent Gram-negative bacterial order of the human gut, encode at least one T6SS (Coyne et al., 2016). Finally, a recent report shows that the antibacterial activity of *Bacteroidetes fragilis* is active in the mice gut and that it kills several members of the microbiota *in vitro*, suggesting a role in the gut colonization (Chatzidaki-Livanisa et al., 2016). Altogether, the T6SS antibacterial activity clearly has a role in the eukaryotic host as well, and should be studied into more details.

**Figure 1:**
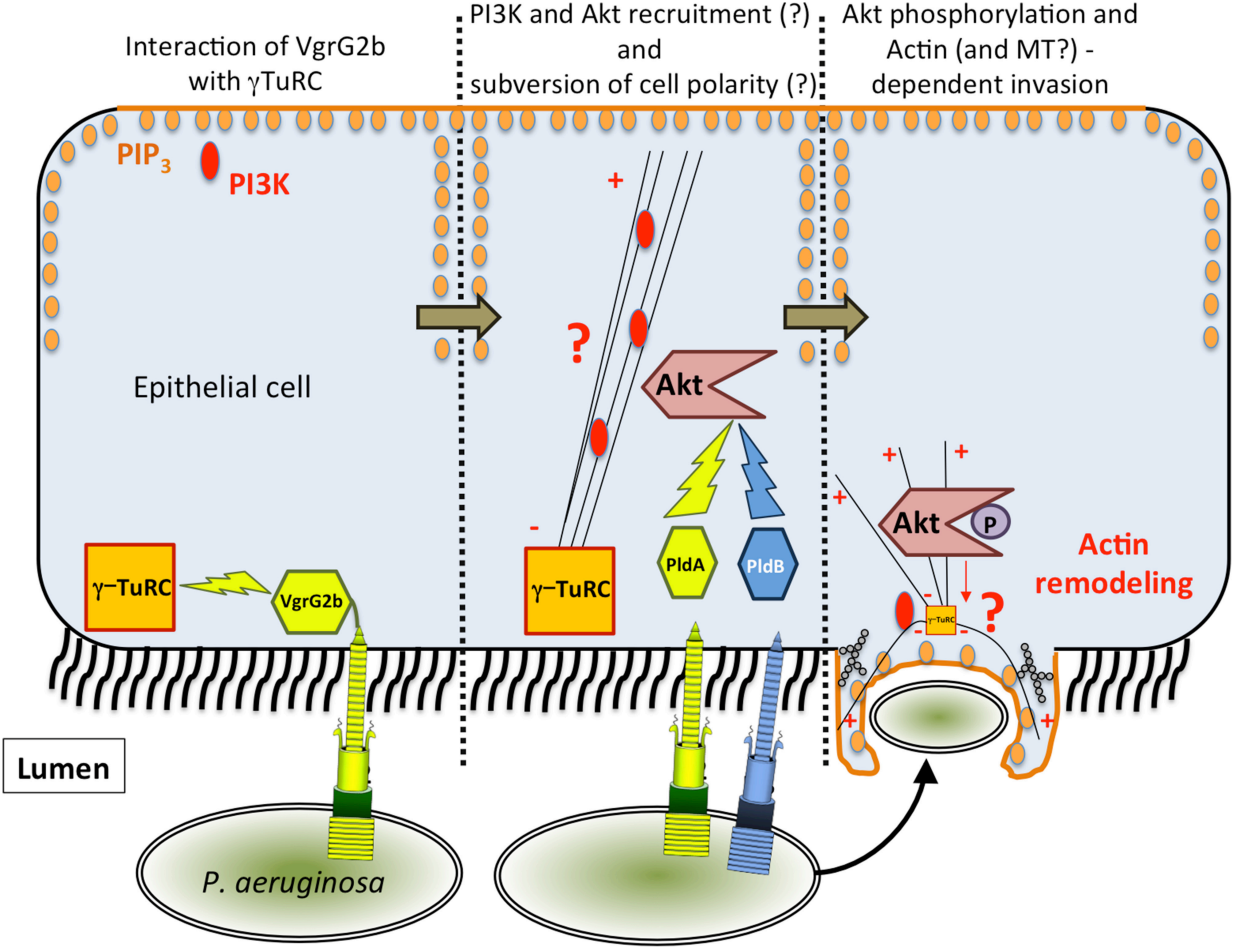
**Model of T6SS-dependent internalization of *P. aeruginosa* into epithelial cells**. In the proposed model, VgrG2b targets γ-TuRC, which could lead to the recruitment of PI3K at the apical membrane. Then, PldA/B both target Akt leading to actin remodeling and finally to the entry of *P. aeruginosa*.

Despite being considered an extracellular pathogen, several reports demonstrate that *P. aeruginosa* actively invades non-phagocytic cells, such as the epithelial cells that line the mucosal barrier and the endothelial cells that form the vascular lumen (Chi et al., 1991; Engel and Eran, 2011). The entry step requires the actin network, most probably to allow membrane protrusion (Fleiszig et al., 1995). This is thought to help bacteria avoiding the immune system or to invade deeper tissues during the infection process. Although, bacteria are present in the lumen and therefore at the apical side of the epithelium, *P. aeruginosa* can only internalize through membrane that displays basolateral characteristics (Figure 1). To circumvent this, *P. aeruginosa* is able to transform apical membrane into basolateral membrane, creating a local microenvironment that facilitates colonization and entry into the epithelium (Kierbel et al., 2007). Interestingly, *P. aeruginosa* is also able to transmigrate through an epithelial barrier, taking advantage of cell division sites and senescent cell extrusion (Golovkine et al., 2016). Altogether, these convergent mechanisms for entering or crossing the epithelial barrier suggest that this ability is essential for successful colonization of the host by *P. aeruginosa*.

The mechanism by which *P. aeruginosa* recruits host factors to internalize within non-phagocytic cells is still poorly understood. Among the various factors required for this process (for a review see Engel and Eran, 2011), we demonstrated that the H2-T6SS machinery (Figure 1) promotes the uptake of *P. aeruginosa* into pulmonary epithelial cells but, at the time, the identity of the cognate effector(s) involved remained to be discovered (Sana et al., 2012). Two recent reports have enabled key new insights into the T6SS-mediated invasion mechanism of *P. aeruginosa*. Host-cell invasion requires two phospholipase D enzymes, PldA and PldB, which are injected via the H2-T6SS or H3-T6SS machineries, respectively (Jiang et al., 2014; Table 1). The H3-T6SS machinery is thus required for *P. aeruginosa* internalization. PldA and PldB both target the host PI3K (phosphoinositide 3-kinase)/Akt pathway, which is hijacked during the internalization process (Kierbel et al., 2005; Engel and Eran, 2011). After injection into epithelial cells, the two T6SS effectors were shown to directly bind Akt, which may lead to activation of the PI3K-Akt signaling pathway. Indeed, Akt phosphorylation is thought to promote a profound remodeling of the apical membrane in which protrusions enriched in PIP_3_ (phosphatidylinositol-3,4,5-triphosphate) and actin form, facilitating further entry of *P. aeruginosa* (Bleves et al., 2014; Jiang et al., 2014). Interestingly, PldA and PldB are also known to target bacterial cells, making them trans-kingdom effectors (Bleves et al., 2014). Host-cell invasion also requires the evolved VgrG2b effector (Sana et al., 2015). VgrG2b is injected via the H2-T6SS into epithelial cells where it targets the microtubule network and more interestingly the gamma-tubulin ring complex components (γ-TuRC) of the microtubule nucleating-center (Kollman et al., 2011; Table 1). Remarkably this interaction is followed by a microtubule-dependent internalization of the pathogen since treatment of epithelial cells with drugs that disrupt the microtubule network decreases the number of internalized bacteria. Furthermore, injection of VgrG2b via the H2-T6SS machinery can be bypassed by directly producing VgrG2b in epithelial cells prior to infection. This can even lead to the internalization of H2-T6SS or *vgrG2b* mutants suggesting that VgrG2b is a central player in this process.

How can microtubule and actin cytoskeletons be integrated in a common invasion process? In Figure 1 we propose a working model that is restricted to the internalization mediated by the T6SS effector interplay. As mentioned above, the internalization of *P. aeruginosa* is a multifactorial process, and our goal here is to integrate and discuss the functions of the three anti-eukaryotic T6SS effectors encoded by the *P. aeruginosa* genome. H2-T6SS first injects VgrG2b which targets the microtubule network and in particular γ-TuRC. This interaction could subvert the polarization of epithelial cells by creating novel sites of non-radial microtubule nucleation along the apical-basal axis at the bacterium-binding site. These new sites would be enriched in microtubule-minus ends, which might interfere with the directional transport of microtubule-dependent cargoes in the cell among them the basal PI3K marker. Concomitantly, *P. aeruginosa* may also recruit Akt via the PldA and PldB effectors injected by the H2 and H3-T6SS machineries respectively. Indeed the apical PI3K may lead to PIP_3_ synthesis and recruitment of Akt creating a basolateral environment at the apical surface (Figure 1). This will activate the Akt signaling, allowing actin-dependent membrane protrusion, and ultimately the internalization of *P. aeruginosa* into epithelial cells (Figure 1). One can also propose that these protrusions may also contain microtubules. In this model, both H2 and H3-T6SS are essential components for the internalization process of *P. aeruginosa* into epithelial cells. We propose that H2-T6SS is active before H3-T6SS because (i) transcriptional studies show that it is expressed earlier in the growth phase (Sana et al., 2013), (ii) ectopic synthesis of VgrG2b inside epithelial cells trigger internalization of T6SS mutants (Sana et al., 2015), and (iii) PldA and PldB can compensate for each other during infection with stationary-phase grown bacteria (Jiang et al., 2014). However, the exact molecular mechanism by which VgrG2b acts on the γ-TuRC and the microtubule network has yet to be deciphered. Also, what is the nature of the effector domain of the evolved VgrG2b? How does the interaction of Akt with these two phospholipases D trigger its activation? Finally, the intracellular lifestyle of *P. aeruginosa* has to be studied in greater detail, particularly in light of very interesting reports which propose that *P. aeruginosa* creates its own bleb-niche in epithelial cells where it can replicate (Angus et al., 2008; Jolly et al., 2015).

More efforts have to be made to decipher this entire mechanism because it could lead to important biomedical applications. Indeed, *P. aeruginosa* is known to induce acute infection in patients with burned skin. Rationally, in this scenario, the first barrier *P. aeruginosa* will have to cross will be the skin, which is basically composed of epithelial cells. We also know that H2-T6SS and H3-T6SS are important for full virulence in worm models as well as in mouse models (Lesic et al., 2009; Sana et al., 2013). Therefore, it begs the question as to whether H2 or H3-T6SS are responsible for pathogen entry through the burned skin barrier. It will therefore be very interesting over the next years to study this invasion mechanism more deeply using for example a three dimensional model of burned skin (Shepherd et al., 2009). Thus, H2- and H3-T6SS of *P. aeruginosa* are potentially good candidates for new therapeutic targets. And finally, although most invasive bacteria manipulate host actin for entry (Cossart and Sansonetti, 2004) this T6SS-mediated entry mechanism could be common in other pathogens such as *Campylobacter jejuni, and Citrobacter freundii, Neisseria gonorrhoeae, or Burkholderia cepacia* that also appear to modulate the microtubule network to invade epithelial cells (Donnenberg et al., 1990; Oelschlaeger et al., 1993; Grassme et al., 1996; Yoshida and Sasakawa, 2003; Taylor et al., 2010).

## Conclusions

The T6SS machineries of *P. aeruginosa* must be considered as versatile weapons that are able to target both prokaryotic and eukaryotic cells. In the future, studies should aim at determining the role of their antiprokaryotic activity *in vivo* because H1-T6SS is clearly active in cystic fibrosis patients. One could also ask whether this T6SS-driven antibacterial activity is a common weapon used by pathogens *in vivo* to outcompete either the commensal microbiota or other pathogens. As shown in Table 1 the repertoire of T6SS effectors in *P. aeruginosa* may not be complete and at least 4 H2-T6SS putative effectors can be proposed according to their genetic linkage with known effector genes (Table 1). Also, the exact mechanism of T6SS-dependent internalization within epithelial cells should be studied in more detail and its role in colonization and pathogenicity should be better understood.

## Author contributions

TS and SB wrote the review and created Figure 1. BB and SB created Table 1.

## Funding

TS was financed by a Ph.D. fellowship from the French Research Ministry and with a “Teaching and Research” fellowship from AMU. This work is supported by a grant (N°RF20150501346/1/69) from “Association Gregory Lemarchal” and “Vaincre la Mucoviscidose” and by AMU and CNRS.

### Conflict of interest statement

The authors declare that the research was conducted in the absence of any commercial or financial relationships that could be construed as a potential conflict of interest.
